# Setmelanotide: A Melanocortin-4 Receptor Agonist for the Treatment of Severe Obesity Due to Hypothalamic Dysfunction

**DOI:** 10.17925/EE.2024.20.2.9

**Published:** 2024-02-09

**Authors:** Sulmaaz Qamar, Ritwika Mallik, Janine Makaronidis

**Affiliations:** 1. Centre for Obesity Research, Rayne Institute, Department of Medicine, University College London, London, UK; 2. UCLH Bariatric Centre for Weight Management and Metabolic Surgery, University College London Hospital, London, UK; 3. National Institute of Health Research, UCLH Biomedical Research Centre, London, UK; 4. Department of Diabetes and Metabolism, Barts Health NHS Trust, London, UK

**Keywords:** Hypothalamic dysfunction, leptin–melanocortin signalling pathway, melanocortin-4 receptor agonist, monogenic obesity, setmelanotide, syndromic obesity

## Abstract

Obesity is a silent global pandemic. It is a condition associated with multiple risk factors and adverse outcomes that arise from the intertwined relationship between environmental factors and genetics. The genetic factors that cause phenotypic expression are variable. Monogenic obesity is a severe early-onset and rarer form of obesity, which presents with co-morbidities such as abnormal feeding behaviour. Monogenic obesity causes impaired weight regulation in the hypothalamus due to defects in the leptin–melanocortin signalling pathway. The emergence of a new therapeutic treatment, the melanocortin-4 receptor agonist setmelanotide (originally RM-493), has represented a breakthrough in the management of monogenic obesity and has raised hope in managing complex obesity. This review provides an overview of the setmelanotide trials that have taken place, as well as its mechanism of action, side effects and weight loss outcomes that led to its approval in the treatment of pro-opiomelanocortin (POMC) deficiency and proprotein convertase subtilisin/kexin type 1 (PCSK1) deficiency. It also explores setmelanotide's role in other genetic forms of obesity, such as hypothalamic obesity, Prader-Willi syndrome, Alström syndrome and other rare genetic conditions that are being investigated. This review aims to help to understand the pathophysiology of genetic obesity and aid in future treatment options for people with severe, complex genetic obesity.

Obesity defined as abnormal or excessive fat accumulation that presents a risk to health, is a chronic disease linked to metabolic co-morbidities, such as type 2 diabetes mellitus and cardiovascular disease, a reduced life expectancy, economic burden and reduced quality of life.^1^ Worldwide obesity rates nearly tripled from 1975 to 2016, with over 650 million adults (18 years or older) now living with obesity.^2^ The World Obesity Federation predicts that obesity will affect one billion people globally by 2030.^3^

The pathophysiology of obesity is complex, varied and multifactorial.^4,5^ Both genetic and epigenetic factors strongly impact body weight, with up to 70% of BMI variability being heritable. Genetic predisposition has been shown to have a significant impact on energy metabolism, and genetic factors can result in severe early-onset obesity.^6^ Genome-wide association studies have discovered 127 sites in the human genome associated with obesity. The aetiology of genetic obesity is classified into the following categories: monogenic obesity, single gene defects, syndromic obesity and polygenic obesity.^7^ Genetic obesity predominantly originates in the hypothalamic pathways that intercede with leptin's metabolic and behavioural effects and disrupts the leptin–melanocortin pathway, which is the predominant signalling pathway for appetite and satiety.^8^ To develop effective therapeutic interventions for obesity, leading to sustained weight loss and health improvements in the long term, it is critical to have a detailed understanding of the physiological, behavioural and genetic determinants of body weight.

An intact hypothalamic melanocortin pathway is critical for the homeostatic control of energy balance, appetite and body weight, and interruptions to signalling across parts of this pathway have been demonstrated to lead to obesity, often resistant to treatment through energy restriction.^9^ The central hypothalamic melanocortin-4 receptor (MC4R), therefore, constitutes a distinctly validated therapeutic target for the treatment of genetic obesity. Several attempts have been made to develop a specific MC4R agonist to treat obesity; five MC4R agonists have been investigated so far in humans.^9^ Setmelanotide, a selective MC4R agonist, has been shown to induce satiety and lead to reductions in body weight in patients with obesity secondary to rare genetic variants across the pro-opiomelanocortin (POMC)/MC4R pathway.^10^ Its use has been approved by the US Food and Drug Administration (FDA) and the National Institute of Health and Care Excellence (NICE), which provides national guidance and advice in the UK, for certain uncommon monogenic obesity diseases. This review provides a background on the genetic aetiologies for obesity and focuses on setmelanotide, its mechanism of action, and its efficacy as a treatment for obesity secondary to a number of genetic variants in the POMC/MC4R pathway.

## The endocrine regulation of appetite and body weight

In the central nervous system, the hypothalamus integrates peripheral signals of short-and long-term energy availability originating across the gastrointestinal tract and the adipose tissue.^11^ In an energy-deficient state, the orexigenic hormone ghrelin is secreted from the stomach to drive energy intake. In the arcuate nucleus of the hypothalamus, ghrelin acts on neurons expressing neuropeptide Y (NPY) and agouti-related protein (AgRP), generating orexigenic responses and a drive to eat. Once food has been consumed, this is sensed along the gastrointestinal tract; in response to nutrient sensing, a series of hormones are secreted, such as glucagon-l ike peptide 1 (GLP-1), peptide YY (PYY) and oxyntomodulin. These peptides bind to POMC neurons in the hypothalamus. POMC is cleaved by proprotein convertase subtilisin/kexin type 1 (PCSK1) into melanocortin ligands, including α-and β-melanocyte stimulating hormones, which in turn activate the MC4R, which generates a feeling of satiety, improves insulin sensitivity and increases energy expenditure.^12,13^

In addition, the adipokine leptin, which originates from the adipose tissue, is a signal of long-term nutritional state and energy availability. The action of leptin via the leptin receptor (LEPR) also plays a pivotal role in body weight regulation and energy homeostasis.^4^ Leptin stimulates POMC neurons to promote satiety by regulating orexigenic neurons in the arcuate nucleus of the hypothalamus in an opposing way to ghrelin. Leptin deficiency results in hyperphagia and severe early-onset obesity. Notably, a recent report of exogenous leptin treatment in a patient with leptin deficiency highlighted key interactions between gut hormones.^14^ Leptin supplementation resulted in significant rises in meal-stimulated insulin, GLP-1 and PYY levels and a reduction in ghrelin levels, highlighting the regulatory role of this protein.

However, eating constitutes a behaviour, and in an environment where energy availability exceeds requirements, eating is influenced by factors beyond homeostasis. Metabolic and reward-related pathways in central nervous system centres regulating eating behaviour have evolved over thousands of years, predominantly in conditions of sparse energy availability. Interestingly, MC4R mutations leading to increased appetite have been identified in animals adapting to low food availability.^15^ Obesity occurs when energy intake chronically exceeds energy expenditure. At a population level, up to 70% of an individual’s body weight is thought to be genetically determined, with environmental, epigenetic, social and cultural factors determining phenotypic expression.^16^ Beyond genome-derived risk alleles, however, a number of genetic causes of severe early-onset obesity have been identified, accounting for 2% to 5% of severe obesity cases, due to mutations along the hypothalamic POMC/MC4R pathway, which will be discussed in more details in following sections.^17^

## Genetic aetiology of obesity

The prevalence of obesity due to genetic causes varies significantly according to the syndrome, gene and population. Despite the increase in accessibility to genetic testing, genetic causes of obesity remain underdiagnosed. It is estimated that genes involving the melanocortin pathway contribute to approximately 5% of early-onset severe obesity, which is thought to be an underrepresentation; thus, there is a need to maintain a high index of suspicion for accurate diagnosis.^18,19^ Genetic testing is indicated based on clinical suspicion or in cases of severe early-onset forms of obesity.^20^

### Syndromic obesity

So far, between 20 and 30 Mendelian disorders due to discrete genetic defects or chromosomal rearrangement in the form of autosomal dominant, recessive or X-l inked with features of obesity have been identified; these are often observed in conjunction with neurodevelopmental defects with congenital defects or malformations. ^7,21^ The most common forms of syndromic obesity and their respective clinical features with the genes affected are summarized in *Table 1*.^22–27^

### Monogenic obesity

Disruption of the hypothalamic circuits controlling body weight and appetite caused by mutations in the genes encoding leptin, LEPR, POMC and MC4R can lead to severe obesity in humans. Over the past decades, accumulating evidence has described a cluster of genetic disorders presenting with severe obesity alone.^28^ The clinical features and courses of these patients have additionally expanded our understanding of the function of hypothalamic appetite-regulating circuits.^28^ Weight gain and hyperphagia represent the earliest signs of hypothalamic dysfunction, and affected children can demonstrate exponential weight gain, reaching a BMI of >3 standard deviations above the average by the first year of life.^6^

**Figure 1:**
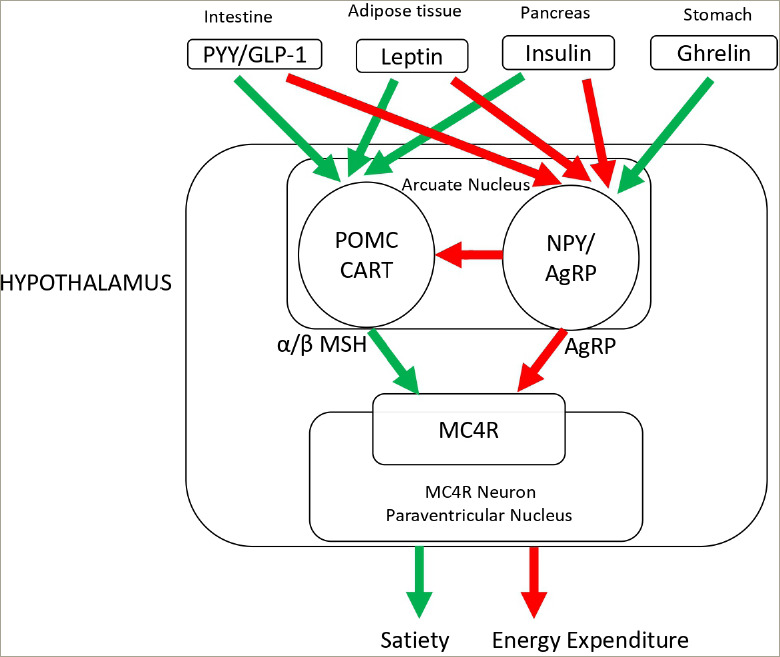
Hypothalamic melanocortin-4 receptor pathway resulting in appetite and weight regulation. Melanocortin-4 receptor pathway defect causes non-syndromic obesity

Disruption at various levels of the leptin–melanocortin pathway or downstream signalling by gene mutations can result in obesity (Figure 1). Genetic defects resulting in monogenic obesity are outlined in *Table 2*.^5,29–31,33,36,37,39,40^

In 1997, *LEP* became the first gene to be associated with monogenic obesity.^30^ The *LEP* gene, previously called the obese (*ob*) gene, is responsible for encoding the protein leptin, which is produced by adipose tissue. Leptin then binds to the LEPR in the arcuate nucleus of the hypothalamus. This binding inhibits orexigenic neurons while upregulating anorexigenic neurons, thus suppressing appetite. *LEP* variants cause congenital leptin deficiency, which is mostly seen in cases of consanguinity. Successful treatment involves the administration of an exogenous leptin called metreleptin.^30^ LEPR deficiency is a rare autosomal recessive disorder caused by pathogenic variants of the LEPR gene (*LEPR*).^39^ Although rare, it is observed to be more prevalent in Europe according to the available literature.^39^ While the symptoms of LEPR deficiency are similar to those observed for *LEP* deficiency, the treatment differs.^39^ Moreover, LEPR deficiency does not respond to treatment with recombinant leptin therapy.^32^

The activation of LEPR stimulates POMC with downstream interaction of the steroid coreceptor activator-1 (SRC1) protein with the phosphorylated form of signal transducer and activator of transcription 3 (STAT3), as well as the Src homology 2 B adaptor protein 1 (SH2B1).^36^ Procovertase enzyme encoded bythe *PCSK1* gene cleaves POMC to alpha melanocyte-stimulating hormone (α-MSH), which stimulates MC4R on neurons expressing the single-minded 1 (*SIM1*) gene in the paraventricular nucleus. Homozygous or compound heterozygous mutations of POMC result in early-onset obesity. In the pituitary, POMC is the precursor for adrenocorticotrophin hormone, which stimulates the release of cortisol and androgens. POMC is also the precursor for the MSH, which produces melanin, the pigment that provides colour to hair and skin. Hence, patients with POMC deficiency can have symptoms of adrenal insufficiency and pale skin, and Caucasians may have red hair due to the absence of melanin.^38^ The phenotype in children from other ethnic backgrounds may not be as evident, as red roots may be present in conjunction with dark hair. Mutations in POMC-derived ligands, which are mentioned below, result in the disruption of the MC4R signalling pathway.

**Table 1:** Most common causes of syndromic obesity

Syndrome	Gene involved/locus	Autosomal dominant/recessive	Clinical features	Estimated incidence
Prader Willi syndrome^22,23^	15q11.2-q13	Autosomal dominant	Hypotonia, hypogonadism, short stature, mental retardation	1/15,000 births
Bardet–Biedl syndrome^22,23^	22 genes have been found	Autosomal recessive	Dysmorphic extremities, mental retardation, hypogonadism, structural anomalies of kidney and renal dysfunction, retinal dystrophy	1/140,000 to 1/60,000 births in North America and Europe and 1/13,500 births in Israel and Arab countries
Alström syndrome^22–24^	*ALMS1* gene	Autosomal recessive	Dysmorphic features, short stature, retinal dystrophy, developmental delay, endocrine abnormalities (hypogonadism, hypothyroidism, PCOS), dilated cardiomyopathy	1/10,000 to <1/1,000,000 births
16p11.2 microdeletion syndrome^22^	Small region chromosome 16	Autosomal dominant	Intellectual disability and developmental delay	1/2,000 births
Cohen syndrome^23,25^	8q22	Autosomal recessive	Microcephaly, ophthalmopathy, prominent central incisors	1/105,000 births (based on Finnish data)
Fragile X syndrome^23,26^	Xq27.3/FMR1	Autosomal dominant	Macro-orchidism, learning difficulties, high-pitched jocular speech	1/4,000 to 1/7,000 births
Albright hereditary osteodystrophy^23,27^	20q13.2/GNAS1	Autosomal dominant	Skeletal defects, short stature, impairment in olfaction	3–4/1,000,000 live births in Japan

*PCOS = polycystic ovary syndrome.*

MC4R variants represent the most common cause of monogenic obesity. More than 170 MC4R variants have been described in the past few decades, and not all of them are pathogenic.^34^ Pathogenic mutations on the MC4R variants are inherited in a co-dependant manner, with a complete loss of function resulting from nonsense mutations or heterozygous frameshift mutations, leading to a more severe phenotype.^33,35^

## Monogenic obesity management or treatment modalities

### Lifestyle and dietary advice

It is crucial to appreciate that individuals with monogenic obesity have impaired hunger–satiety feedback. The absence of satiety signalling limits the success of lifestyle interventions focused on restricting energy intake and increasing physical activity. Hyperphagia can be severe and challenging to control; recommended strategies include restricted food access and established eating routines aimed at reducing impulsivity and food-seeking behaviours.^22^ A clinical study demonstrated that children with MC4R mutations who received lifestyle and psychological interventions for weight management exhibited comparable weight loss to a BMI-matched group of children with obesity without MC4R mutations; however, they were unable to maintain the weight loss compared with their counterparts without MC4R mutation.^41^ The management of people with obesity due to POMC pathway mutations should involve a multidisciplinary, comprehensive and holistic multidisciplinary approach. This includes specialist dieticians and psychologists to design nutritional, behavioural and exercise programmes tailored to the individual.

### Bariatric surgery

Overall, metabolic and bariatric surgery (MBS) in patients with severe obesity is associated with long-term weight loss, increased life expectancy and an overall decrease in mortality compared with conventional treatment.^42,43^ However, animal studies showed that an intact melanocortin system is required for sustained weight loss effects after Roux-en-Y gastric bypass (RYGB).^44^ Data from MBS outcomes in people with monogenic obesity have yielded heterogeneous results depending on the underlying genetic mutation, the type of surgery and the duration of follow-up.^45–50^ For instance, carriers of heterozygous gene variants in the leptin–melanocortin pathway (in seven different genes: *LEPR*, *PCSK1*, *POMC*, *SH2B1*, *SRC1*, *MC4R* and *SIM1*) showed significant weight regain after RYGB in the medium and long term, although they initially demonstrated effective weight loss.^51^ Fifteen years after RYGB, the mean percentage of weight regain after maximum weight loss was 52.7 in carriers compared with 29.8 in non-carriers. The nadir percentage total body weight loss was lower in carriers compared with non-carriers. In a retrospective analysis of 8 people with variants of *LEPR*, *POMC* and *MC4R* who underwent MBS, there was a persistence of hyperphagia, and weight regain occurred with a maximal follow-up of 19 years post-surgery.^52^

### Pharmacotherapy

The first specific treatment for obesity due to a single gene defect for leptin deficiency in the form of recombinant leptin therapy, which reduces appetite and increases satiety, resulting in weight loss, was first approved by the FDA in February 2014.^53,54^ The recombinant analogue of leptin, metreleptin, is an FDA-approved treatment for leptin deficiency due to *LEP-* gene defects, acquired and congenital lipodystrophy. In these patients, metreleptin also improved insulin sensitivity, lipid profiles, and hepatic steatosis and can lead to the normalization of hypothalamic– pituitary axes.^54^ Leptin therapy is not effective in other types of obesity due to the presence of leptin resistance.

*The green arrow indicates a stimulatory effect; the red arrow indicates a inhibitory effect.*

*AgRP = agouti-related regulatory peptide; CART = cocaine-and amphetamine-regulated transcript; GLP-1 = glucagon-like peptide-1; MC4R = melanocortin-4 receptor; NPY = neuropeptide Y; POMC = pro-opiomelanocortin; PYY = peptide YY; α-MSH = alpha melanocyte-stimulating hormone; β-MSH = beta melanocyte-stimulating hormone.*

### Melanocortin receptor agonism

Melanocortin receptors, as they are G protein-coupled receptors, have been recognized as a therapeutic target due to their distribution in multiple tissues (i.e. skin, adrenal glands, exocrine glands, immune cells and brain) and their ability to control other physiological processes, such as cortisol release, inflammation, secretions, satiety and pigmentation.^55^ There have been several attempts to develop melanocortin agonists of different melanocortin receptors; however, the fourth agonist was identified as a potential treatment option. LY2112688, a first-generation MC4R agonist, was associated with increased heart rate and significant increases in systolic and diastolic blood pressure, which was sustained for the duration of the treatment.^56^ In addition, increased muscle stiffness, yawning, stretching and penile erections were reported. Bremelanotide, an active metabolite of Melanotan-I I, had also been investigated; however, it was also associated with a series of adverse effects, such as cardiovascular instability, hyperpigmentation and spontaneous penile erection in male patients.^9,57,58^ It eventually received approval for acquired hypoactive sexual desire disorder in pre-menopausal women.^59^

## Setmelonatide

Setmelanotide (IMCIVREE®; Rhythm Pharmaceuticals, Inc., Boston, MA, USA), an eight-amino-acid cyclic peptide previously known as BIM-22493 or RM-493, is a novel, synthetic, highly-selective MC4R agonist.^9^ In a non-human primate model of diet-i nduced obesity, there was a reduction in food intake and persistent weight loss.^60^ Following landmark trials, outlined in *Table 3*,^61–64^ setmelanotide initially received marketing authorization from the FDA in 2020 for the treatment of monogenic or syndromic obesity in adults and children over 6 years of age due to defects that impair the MC4 pathway, such as patients with POMC, PCSK1 and LEPR deficiencies.^65^ Later, it received FDA approval for use in Bardet–Biedl syndrome (BBS).^66^ The European Medicines Agency has also approved setmelanotide for people with severe obesity due to POMC or LEPR deficiency and BBS.^67^ In the UK, NICE guidance recommends setmelanotide for POMC and LEPR deficiency for ages 6 and above, and it is only recommended if the company provides it according to the commercial arrangement.^68^ Its role is being investigated in other genetic forms of obesity due to Prader-Willi syndrome, Alström syndrome (AS), and in hypothalamic obesity. Prior to this, there has been no available targeted therapy for patients with obesity due to monogenic obesity.

**Table 2:** Genetic defects resulting in monogenic obesity

Genetic defect	Prevalence	Clinical features/phenotypes	Mutation(s)
*LEP* deficiency^5,29–32^	More prevalent where consanguinity is found and <1,000 cases identified worldwide	Hyperphagia Lack of satiety Delayed puberty due to hypogonadotropic hypogonadism Hypothyroidism Increased risk of infections	Homozygous
*LEPR* mutation^5,29–32^	3% with severe-onset obesity	Severe Hyperphagia within 1 week Delayed puberty due to hypogonadotropic hypogonadism Increased bone mineral density Altered immune response	Compound heterozygous, homozygous
Melanocortin-4 receptor mutation^5,28–35^	3–6% with severe early obesity in BMI >40 kg/m^2^	Hyperphagia Severe hyperinsulinemia Increased linear growth and lean mass	Homozygous, compound heterozygous, heterozygous
POMC deficiency^5,28–32^	Less than 15 diagnosed worldwide	Onset of symptoms within few months of life Hyperphagia Severe early-onset obesity Cholestatic jaundice at infant age Pale skin pigmentation with red hair, adrenal insufficiency and obesity	Homozygous, compound heterozygous
*PCSK1* deficiency^5,29,31,32^	<20 cases worldwide	Moderate hyperphagia Central hypothyroidism ACTH deficiency causing adrenal insufficiency Postprandial hypoglycaemia Hypogonadotropic hypogonadism Diabetes insipidus Intestinal dysfunction	Homozygous, compound heterozygous
Neurotrophic tyrosine kinase receptor type 2 mutation^5,29,32^	<10 cases identified worldwide	Hyperphagia Developmental delay Nociception anomalies Short-term memory impairment	Compound heterozygous
*SIM1* loss of function/deficiency^5,28–31^	Not available	Hyperphagia Severe Obesity Low systolic blood pressure Speech and language delay Autistic behaviour	Heterozygous
*ADCY3* ^29^	Not available	Defects in olfaction Mild intellectual disability	Autosomal recessive 2p23
BDNF^5,29,31,32^	Not available	Hyperphagia Hyperactivity Intellectual disability	Autosomal dominant deletion 11p14
*SRC1* deficiency^31,36^	Not available	Hyperphagia Partial thyroid hormone resistance Menorrhagia Adipose tissue fibrosis Hepatic fibrosis	Heterozygous variants
*SH2B1* deficiency^28,29,31,32,37,38^	Not available	Hyperphagia Variable developmental delay Insulin resistance	Microdeletions in chromosome 16p11.2

*ACTH = adrenocorticotropic hormone; ADCY3 = adenylate cyclase 3; BDNF = brain-derived neurotropic factor; BMI = body mass index; LEP = leptin; LEPR = leptin receptor; PCSK1 = proprotein convertase subtilisin/kexin type 1; POMC = pro-opiomelanocortin; SH2B1 = Src-homology-2 B adaptor protein 1; SIM1 = single-minded 1; SRC1 = steroid receptor co-activator 1.*

### Mechanism of action

**Figure 2:**
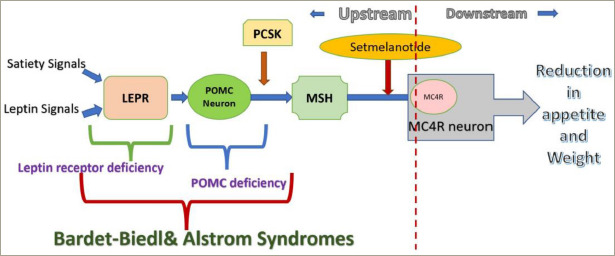
Mechanism of action of setmelanotide

When administered peripherally, setmelanotide crosses the blood–brain barrier and acts on the paraventricular nucleus of the hypothalamus and lateral hypothalamic area, the areas associated with appetite regulation. By binding to MC4R, it replaces the missing link in the hypothalamic leptin–melanocortin pathway, resulting in appetite suppression (Figure 2) and improved insulin resistance.^61^ It also results in increased resting energy expenditure by shifting substrate oxidation to fat.^69^

Apart from MC4R activation, it also activates multiple other melanocortin receptors, such as MC3R (regulates immune function), MC1R (regulates pigmentation and immune function), MC5R (regulates sebaceous gland activity) and MC2R (regulates adrenal gland function), with decreased selectivity for the latter two.^9,70^ While first-generation MC4R agonists increased sympathetic tone, resulting in an increase in heart rate and blood pressure, uniquely, setmelanotide, a second-generation MC4R agonist, does not seem to have this effect.^9^ Another unique feature of setmelanotide is that it induces nuclear factor of activated T cell (NFAT) activation and restoration of this signalling pathway for selected MC4R variants. NFAT proteins are associated with immune function and have also been detected in adipocytes, and NFAT signalling appears to be impaired in obesity.^71^ Hence, there is potential for setmelanotide in the treatment of people with diverse MC4R-related pathway deficiencies.^62^ Impairment of cilial signalling in the MC4R pathway has been hypothesized to contribute to obesity in patients with BBS, resulting in leptin resistance; consequently, the potential of setmelanotide to restore MC4R signalling has been investigated.^64^ In a phase Ib trial, setmelanotide results in weight loss in patients who are obese and with MC4R deficiency.^72^ This finding prompted the need for further trials to study the clinically significant weight loss in patients with MC4R deficiency.

**Table 3:** Key trials of setmelanotide

Type of study (year)	Genetics, mean age (SD)	Setmelanotide	No. of patients/duration of treatment received	Main outcomes
Phase II (2016)^61^	POMC deficiency, ages 21 and 26 years	Maximum daily dose of 1.5 mg (initial dose was 0.25 mg and 0.5 mg for the first and second patient respectively)	N=2, 12 weeks	The first patient lost 25.8 kg (16.6%) at 13 weeks and continued with a total weight loss of 51.0 kg after week 42. The second patient lost 20.5 kg (13.4%) at week 12. Likert hunger scale decreased to 0–1 for both.
Phase II (2018)^62^	*LEPR* deficiency, ages 23, 22 and 14 years	Maximum daily dose of 1.5 mg for the first patient and 2 mg for the remaining two patients	N=3, 26 weeks	The first patient lost 25.1 kg (19.2%) at 61 weeks. The second patient lost 13.9 kg (11.4%) at week 36. The third patient lost 10.0 kg at week 13; a dosing error had occurred. Likert hunger scale decreased to 0–1, 2 and 5, respectively
Phase III (2020)^63^	POMC deficiency, 18.4 (6.2) years	Maximum daily dose of 3 mg (initial dose was 0.5 mg in children and 1 mg in adults)	N=10, 52 weeks (including 4 weeks of placebo)	At around 1 year, 80% (8) of patients had a minimum 10% weight los; % weight change (SD) from baseline was -25.6% (9.9) (p<0.0001); absolute % change in most hunger scale was 2.2
Phase III (2020)^63^	*LEPR* deficiency, 23.7 (8.4) years	Maximum daily dose of 3 mg (initial dose was 0.5 mg in children and 1 mg in adults)	N=11, 52 weeks (including 4 weeks of placebo)	At around 1 year, 45% (5) of participants had a minimum of 10% weight loss; % weight change from baseline was -12.5% (8.9) (p<0.0001); absolute % change in most hunger scale was 3.1
Phase III (2022)^64^	BBS and AS, 19.8 (10.2) years	Doses titrated up to a maximum of 3 mg daily	N=38 (32 with BBS and 6 with AS), initial 14 week double-blind period followed by a 52 week open-l abel period	At 52 weeks, in 32.2% of participants with BBS a minimum of 10% weight loss with setmelanotide. For ages 12 years and older with BBS, % weight change from baseline (SD) was -6.5% (7.0) (p<0 .0001); absolute % change in maximal hunger scale is -30.5% (26.5); p=0·0004. For patients with AS, results were inconclusive

*AS = Alström syndrome; BBS = Bardet–Biedl syndrome; LEPR = leptin receptor; N = total population; No. = number; POMC = pro-opiomelanocortin; SD = standard deviation.*

*The blue arrow indicates a stimulatory effect; the red arrow indicates a inhibitory effect.*

*LEPR = leptin receptor; MC4R = melanocortin-4 receptor; MSH = melanocyte-stimulating hormone; PCSK = proprotein convertase; POMC = pro-opiomelanocortin.*

### Phase II studies

A number of phase II and III clinical trials have been carried out to date. *Table 3* highlights three phase III trials led by Rhythm Pharmaceuticals and two phase II investigator-l ed trials.

In a pilot phase II study (ClinicalTrials.gov identifier: NCT02507492), two patients with POMC deficiency were treated with setmelanotide.^61^ The patients experienced not only a sustainable reduction in hunger but also significant weight loss (51.0 kg at week 42 in the first patient and 20.5 kg at week 12 in the second patient). Although there were only two patients in this study, the results were impressive, as there appeared to be a complete reversal of hyperphagia, impressive weight loss, correction of insulin resistance and significant improvement in quality of life. It was then hypothesized that defects in LEPR signalling, resulting from impaired activation of POMC neurons due to *LEPR* gene mutations, might similarly contribute to a lack of MSH signalling. Thus, treatment with an MC4R agonist might be beneficial. In another investigator-initiated phase II trial (ClinicalTrials.gov identifier: NCT02507492), three people with severe obesity due to LEPR deficiency were administered setmelanotide.^62^ The results of this trial indicate significant and sustained reductions in hyperphagia and body weight, which were sustained over the observation period of 45–61 weeks. This finding opened up new avenues for the treatment of individuals with LEPR deficiency and additional patient populations with genetic deficiencies (*LEP*, *POMC*, *PCSK1*, *MAGEL2*, *CPE*) that lead to the defective functioning of the MC4R pathway.

In the phase II trial (ClinicalTrials.gov identifier: NCT03013543), setmelanotide resulted in the reduction of body weight and hyperphagia (assessed by hunger scores) after 3 months in patients with heterozygous POMC mutations, including PCSK1, LEPR, SRC1 and SH2B1 variants.^73^ It was reported that the following patients achieved ≥5% weight loss after 3 months of setmelanotide treatment: 12/35 (34.3%) patients with heterozygous POMC, PCSK1 or LEPR variants; 9/30 patients (30.0%) with heterozygous SRC1 variants; and 13/35 (37.0%) patients with heterozygous SH2B1 variants. The mean percentage change in body weight was -10.1% for responders and -0.4% for non-responders.^73^ A phase II trial was undertaken to investigate the efficacy of setmelanotide over 12 months for treating obesity and hunger, as well as cardio-metabolic outcomes, in patients with BBS (ClinicalTrials.gov identifier: NCT03013543).^74^ The mean percentage change in weight from baseline to 3 months was -5.5% (n=8), at 6 months was -11.3% (n=8) and at 12 months was 16.3% (n=7). Setmelanotide was well tolerated and reduced body weight and hunger in participants with BBS with no concerns about safety.

Additionally, there are a number of phase II clinical trials currently underway that aim to evaluate the safety and efficacy of setmelanotide for different indications. A phase II clinical trial with setmelanotide in patients with rare genetic disorders of obesity has completed recruitment (ClinicalTrials.gov identifier: NCT03013543).^75^ It involves investigating once-daily setmelanotide injections in patients ≥6 years across various cohorts of patients with several defects in the MC4R pathway. Furthermore, the DAYBREAK study (ClinicalTrials.gov identifier: NCT04963231) is a double-blind, placebo-controlled, phase II study of setmelanotide in patients with specific gene variants in the MC4R pathway.^76^

The efficacy and safety analysis of setmelanotide in the management of hypothalamic obesity as a result of hypothalamic injury and impaired MC4R pathway signalling is also being investigated. Interim results of a phase II study of setmelanotide in hypothalamic obesity in patients aged ≥6 have been made available (ClinicalTrials.gov identifier: NCT04725240).^77^ The dose of setmelanotide dose was titrated to a maximum of 3 mg once daily, followed by a maintenance period. Among the 18 patients included, 88.9% achieved a ≥5% reduction in BMI, with a mean 14.9% reduction in BMI across all patients, and 72.2% experienced a ≥10% reduction at 16 weeks. These promising results warrant continued evaluation of setmelanotide in patients with hypothalamic obesity, for whom there is no approved therapy.

### Phase III studies

The main clinical trial evidence to support the use of setmelanotide for the treatment of obesity and hyperphagia caused by POMC or LEPR deficiency was provided by two multicentre phase III trials conducted in North America and Europe.^78^ The POMC trial (ClinicalTrials.gov identifier: NCT02896192) and the LEPR trial (ClinicalTrials.gov identifier: NCT03287960) included participants aged ≥6 years with obesity caused by POMC and LEPR deficiency, respectively. Participants initially received open-l abel setmelanotide for 12 weeks and continued to receive the total study treatment for 52 weeks. The maximum dose was 3 mg, with an initial dose of 0.5 mg for children and 1 mg for adults (≥18 years). In the POMC trial, 10 participants were enrolled, 8 (80%) of whom achieved at least 10% weight loss compared with baseline after 1 year. In the LEPR trial, 11 participants were enrolled, 5 (45%) of whom achieved at least 10% weight loss compared with baseline after 1 year. The mean percentage change in the most hunger score was -27.1% in the POMC trial and -43.7% in the LEPR trial. Compared with first-generation MC4R agonists, setmelanotide did not result in increases in heart rate or blood pressure. Due to the rarity of POMC and LEPR deficiencies, these studies had a small sample size, resulting in limited statistical power and a lack of randomization. Furthermore, hunger was assessed using a Likert-type scale, as no validated hunger scoring system is available for rare genetic disorders of obesity. A sub-study evaluating the improvement in the quality of life reported that setmelanotide improved quality of life as early as week 5, with some participants no longer experiencing impaired quality of life at week 52.^79^ The reason for the improvement in quality of life was attributed to a reduction in hunger and body weight. This study demonstrates the burden of these diseases and the importance of additional psychological support to patients and families through interdisciplinary support.

In a study to evaluate the historical weight trajectory in the patients enrolled in the above trials, 17 patients were included in the analysis.^13^ Prior to setmelanotide treatment, patients were ≥95th percentile for weight, had progressive weight gain and did not respond to weight loss interventions. Setmelanotide resulted in a decrease in weight and BMI trajectories over 1 year. Furthermore, there is now an extension trial for up to 5 years to study the safety and tolerability of continued setmelanotide in patients with obesity associated with genetic defects upstream of the MC4R in the leptin–melanocortin pathway.^80^

The first treatment targeting BBS to be approved was investigated in a multicentre, randomized, double-blind, placebo-controlled, phase III trial (ClinicalTrials.gov: NCT03746522).^64^ Thirty-eight patients were randomized to receive either setmelanotide 3 mg subcutaneous injection daily (n=19) or placebo (16 patients with BBS and 3 with AS in each group) during the 14-week double-blind period, followed by open-l abel setmelanotide for 52 weeks. The results revealed that setmelanotide was associated with significant reductions in weight for patients with BBS; consequently, the first drug treatment for obesity in patients with BBS was approved.^81^ However, these results were inconclusive for patients with AS. In 2022, the FDA provided the Breakthrough Therapy Designation to setmelanotide for hypothalamic obesity.^82^ A phase III trial of setmelanotide in patients with acquired hypothalamic obesity aged 4 years or above is currently underway.^83^ A trial comparing the daily and weekly formulations of setmelanotide in patients with gene defects in the MC4R pathway is also underway.^84^

EMANATE is a phase III trial comprising five independent sub-studies based on genetic variants that is currently underway (ClinicalTrials.gov identifier: NCT05093634).^85^ Participants must have a heterozygous genetic variant in POMC or PCSK1, LEPR, and SRC1 or SH2B1 obesity. Patients are being randomized to receive daily setmelanotide or placebo for 52 weeks with mean change in body weight as the primary outcome. It has an estimated enrolment of 560 patients. If successful, setmelanotide can be used in an expanded population of people with obesity and with other rare genetic diseases.

From the studies described, it is evident that there is a varied response to setmelanotide depending on the underlying pathophysiology. Overall, patients with POMC deficiency appear to respond the best to setmelanotide, followed by patients with LEPR deficiency and patients with BBS. The patients studied had homozygous or compound heterozygous variants of POMC, PCSK1 or LEPR deficiency; however, the effect of certain other heterozygous POMC variants in obesity is still elusive, and more in-depth phenotyping of each heterozygote may be helpful.^86^ Despite the variations reported, setmelanotide is a viable option for patients with genetic obesity, for which it has been approved.

### Adverse effects

Overall, setmelanotide appears to be well tolerated, with injection site reactions (96%) (usually mild) and hyperpigmentation (78%) being reported as the most common adverse effects.^64,78,87^ Skin hyperpigmentation can occur due to off-target activation of MC1R, which is expressed in melanocytes and leads to the accumulation of melanin.^88^ Setmelanotide can result in skin tanning, with darkening of the lips and naevi, and occasional hair colour darkening in patients who are POMC deficient and LEPR deficient; however, no malignant skin changes have yet been observed.^88^ However, it demonstrates the importance of regular skin examination prior to and during the treatment period to rule out any underlying malignancy.

Other adverse events included dry mouth, headache (41%) and gastrointestinal side effects, such as nausea (56%), vomiting (30%) and diarrhoea (37%).^61,78,87^ Disturbance in sexual arousal and spontaneous penile erections (23%) were also reported.^89^ No treatment-related cardiovascular effects, such as the changes in heart rate or blood pressure seen with first-generation MC4R agonists, have been reported.^61^ One possible treatment-related adverse event is hypereosinophilia, although the mechanism responsible for this occurrence remains unclear.^61^ While patients with psychiatric disorders such as moderately severe and severe depression were excluded from the trials, the phase III trials did not report any worsening of depressive symptoms with setmelanotide treatment.^4^ However, setmelanotide may cause depression and suicidal ideation and should be discontinued in case of new-onset or worsening depression.^90^ In a pooled analysis, the onset of adverse events was noted to be higher during the first month of treatment, with fewer events occurring during the subsequent months.^89^

While hypothalamic POMC deficiency may result in improved glucose tolerance, treatment with setmelanotide should, in theory, have the opposite effect; however, setmelanotide did not result in a significant worsening of glucose metabolism parameters in individuals with POMC or LEPR deficiency.^78^ Indeed, setmelanotide was associated with a significant improvement in fasting glucose values in patients with POMC; however, this was not the case for LEPR deficiency. Setmelanotide is not approved for use in neonates and infants due to serious and fatal adverse reactions, including ‘gasping syndrome’, which occurs when neonates and low birth weight infants are treated with benzyl alcohol-preserved drugs.^91^

## Future directions

Although setmelanotide has been approved only for rare genetic forms of obesity, due to its role in reducing leptin resistance its potential effectiveness in non-genetic forms of obesity is still under investigation.^61^ In addition, as our understanding of the genetics of appetite-regulating pathways improves, further indications for setmelanotide treatment may emerge. Once its role in clinical practice has been established and long-term data become available, determining its impact on cardiovascular outcomes will also be important.

Furthermore, while combination approaches are becoming increasingly common in trials for obesity pharmacotherapy, the efficacy and safety of setmelanotide therapy in combination with other pharmacotherapy agents for the treatment of obesity will also need to be established. Interestingly, the combination of the GLP-1 agonist liraglutide and setmelanotide in mice showed improvement in glycaemic control, cholesterol profile and weight loss compared with monotherapy.^92^

## Conclusions

It is an unprecedented time in the clinical management of obesity, with an improved understanding of the diverse pathophysiology of obesity and a new era emerging in obesity treatment featuring a diverse pipeline of obesity pharmacotherapy. Clinical trial data are promising unprecedented weight reduction and improvement in clinical outcomes for people living with obesity. Our increasing understanding of the genetic contributors to weight and the pathophysiology of obesity also offers an exciting opportunity to devise precision-medicine approaches for the treatment of genetic obesity in a patient population with very complex and unmet health needs. Early data from the use of setmelanotide in clinical trials and post-l icencing for the currently approved indications show considerable promise, suggesting that this novel agent will have a distinct role in future treatment algorithms for people with severe obesity due to melanocortin-pathway dysfunction.
